# 

**Published:** 1892-09

**Authors:** 


CONTENTS.

VOL. 39. No. 9.

| | PAGE. |
|---|---|
| Proper Food for Children | 193 |
| The Human Ear | 197 |
| Skin Diseases | 197 |
| Natural Beauty | 199 |
| The Gulf Stream | 201 |
| Canning Fruit | 203 |
| Sleep | 205 |
| Poverty of the Blood | 206 |
| Heat as a Remedial Agent | 207 |
| The Baby | 208 |
| Ear-ache | 209 |
| Science of Ventilation | 210 |
| Begin the Day Aright | 210 |
| Gout | 211 |
| Care of the Finger Nails | 212 |
| Miscellaneous | 212 |
| Literary | 215 |
| God Help Her | 216 |

INDEX TO ADVERTISEMENTS OF HALF A PAGE AND OVER.

| | PAGE. |
|---|---|
| Christian Life | I |
| Magic Fuel Co. | II |
| Bovinine | IV |
| Ayer's Sarsaparilla | V |
| Hall's Journal Premiums. | VII |
| Washington Improvement Co. | VIII |
| Herba Vita | X |
| Royal Baking Powder | XI |
| Buffalo Lithia Water | XI |

